# Supplementary material for: The interactome of intact mitochondria by cross-linking mass spectrometry provides evidence for coexisting respiratory supercomplexes
Source: Mol Cell Proteomics. 2017 Dec 8;17(2):216–32. doi: 10.1074/mcp.RA117.000470 (PMC5795388; doi:10.1074/mcp.RA117.000470)
Supplement: Supplemental Data [file supp_RA117.000470_133922_0_supp_23978_fzffwf.zip › spectra_annotation/mito_DR_spectra_annotation/191-1-22-1-7-1.pdf]

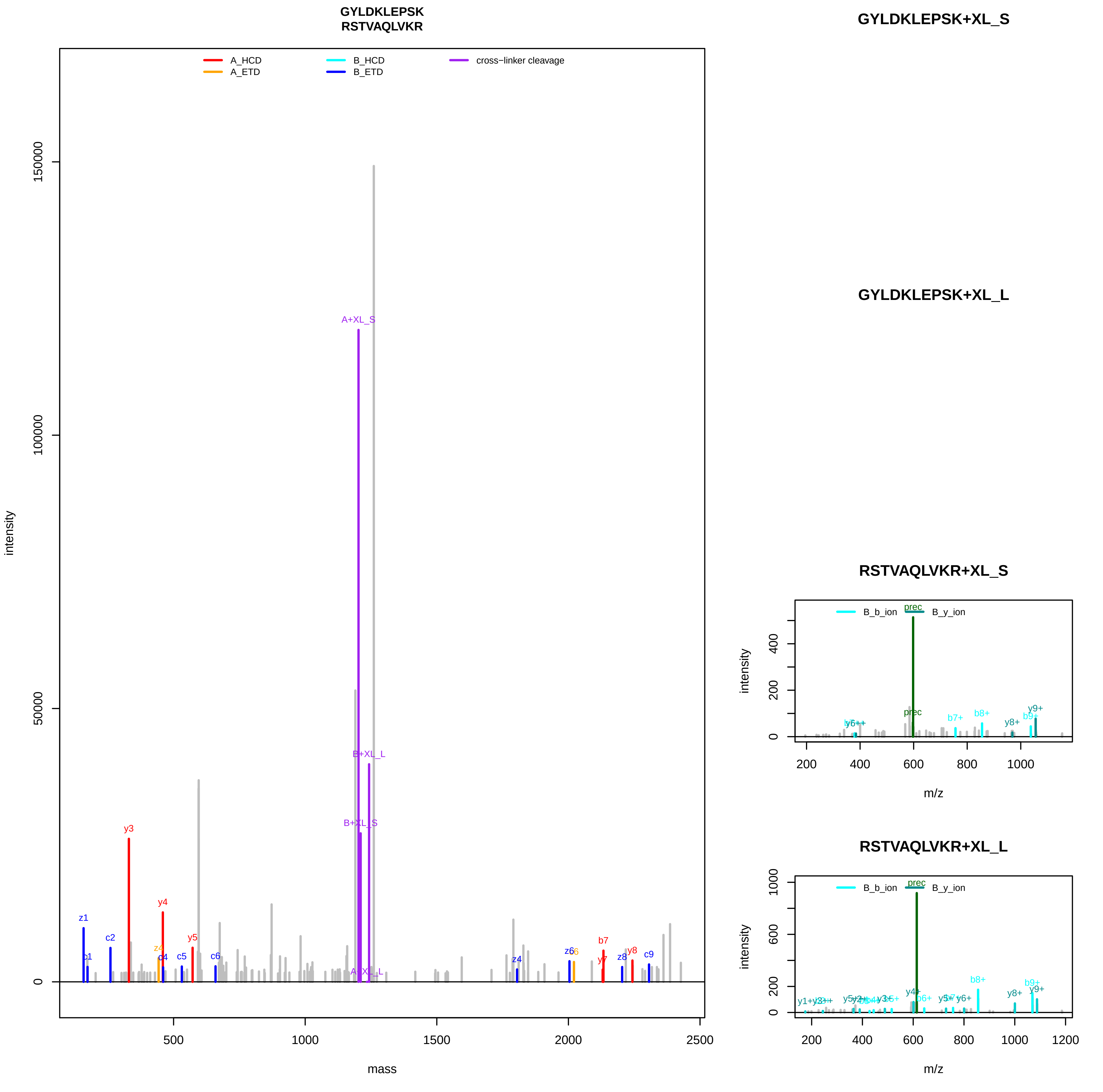
GYLDKLEPSK
RSTVAQLVKR
A_HCD
A_ETD
B_HCD
B_ETD
cross−linker cleavage
A+XL_S
B+XL_L
B+XL_S
A+XL_L
z1
c1
c2
y3
z4
c4
y4
c5
y5
c6
z4
z6
y7
b7
z8
y8
c9
intensity
mass
0
50000
100000
150000
500
1000
1500
2000
2500
GYLDKLEPSK+XL_S
GYLDKLEPSK+XL_L
RSTVAQLVKR+XL_S
B_b_ion
B_y_ion
prec
y6++
b7+
b8+
y8+
b9+
y9+
intensity
m/z
0
200
400
600
800
1000
RSTVAQLVKR+XL_L
B_b_ion
B_y_ion
prec
y4+
b6+
y5+
y6+
b8+
y8+
b9+
y9+
intensity
m/z
0
200
600
1000
200
400
600
800
1000
1200
